# Realistic aspects behind the application of the rat model of chemically-induced mammary cancer: Practical guidelines to obtain the best results

**DOI:** 10.14202/vetworld.2023.1222-1230

**Published:** 2023-06-05

**Authors:** Jéssica Silva, Ana I. Faustino-Rocha, José Alberto Duarte, Paula A. Oliveira

**Affiliations:** 1 Center for the Research and Technology of Agro-Environmental and Biological Sciences (CITAB), Vila Real, Portugal; 2 Institute for Innovation, Capacity Building and Sustainability of Agri-food Production (Inov4Agro), Vila Real, Portugal; 3 Department of Zootechnics, School of Sciences and Technology, University of Évora, Portugal; 4 Comprehensive Health Research Center, University of Évora, Évora, Portugal; 5 Research Center for Physical Activity, Health and Leisure (CIAFEL), Faculty of Sport, University of Porto, Porto, Portugal; 6 Toxicology Research Unit (TOXRUN), Advanced Polytechnic and University Cooperative (CESPU), Gandra, Portugal; 7 Department of Veterinary Sciences, University of Trás-os-Montes and Alto Douro (UTAD), Vila Real, Portugal

**Keywords:** carcinogenesis, guide, modeling, rodents, tips

## Abstract

Cancer is one of the most important public health problems worldwide. Despite the great contribution of *in-vitro* studies for biomedical research, animals are essential to study diseases’ biopathology and diagnosis, and searching for new preventive and therapeutic strategies. Breast cancer is currently the most common cancer globally, accounting for 12.5% of all new annual cancer cases worldwide. Although the rat model of mammary cancer chemically-induced is widely used to study this disease, there is a lack of standardization in procedures for cancer induction, sample collection, and analysis. Therefore, it is important to provide a practical guide for researchers aiming to work with this model to make the analysis of results more uniform. Thus, in this review, we provide the researchers with a detailed step-by-step guide to implement a rat model of mammary cancer, based on our wide experience in this field, to obtain the best results, maximum throughput of each experiment, and easy comparison among researches.

## Introduction

Cancer is one of the most important public health problems worldwide. Despite all the advances in the early diagnosis and treatment of this disease, it is considered the sixth cause of death globally by the World Health Organization (WHO) [1, 2]. According to the same organization, cancer was responsible for 9.8 million deaths in 2020. Consequently, its economic impact is significant, and it is increasing over the years. In 2020, approximately 200 billion U.S. dollars were spent in oncology, including supportive care. An increase of 34% is estimated by 2030, with costs of around 245 billion U.S. dollars, with breast cancer being one of the most frequent cancers among women worldwide. According to the WHO, it victimized approximately 685,000 people in 2020 [3]. These data reinforce the idea that much remains to be done in breast cancer research. Despite the important contribution of *in-vitro* studies for biomedical research, animals have been used by researchers since early times as tools to study human anatomy and physiology, and their diseases [4]. Indeed, animals allow researchers to study many aspects of the diseases, namely their etiology, pathogenesis, progression, genetic and molecular basis, and to develop and evaluate new potential prophylactic and therapeutic strategies that may improve the patients’ quality of life and lifespan [5–7]. Several laboratory animals are available as models for mammary cancer research, namely, mouse, rat, bitch, and cat. In mouse (*Mus musculus*), the murine mammary tumor virus is heavily involved in the genesis of so-called spontaneous mammary tumors, and most tumors are hormone-independent and acinar in origin, which is distinct from human breast cancer. The rat (*Rattus norvegicus*) remains one of the most frequently used animals in laboratory studies in this field [8]. This contributes to the fact that female rats are mammals with many similarities with women, like their anatomy, physiology, genetics, and biochemistry; they are small animals, easily accommodated and manipulated; their physiology and genetic are well known; they are cheaper when compared with other species; their use is approved by the legislation on the protection of animals used for scientific purposes; and they are highly susceptible to chemical carcinogens [6, 9]. It is worth noting that female rats are not susceptible to murine mammary tumor virus. Although the histological and evolutionary characteristics of mammary tumors in bitch [10] and cat [11] are identical to those of breast cancer in women, only spontaneous tumors are studied in these animals, because their experimental manipulation is not possible, for ethical reasons.

There are several models available to study mammary cancer [12]: (i) Laboratory animal strains that spontaneously develop mammary tumors; (ii) implantation of animal or human cancer cell lines in laboratory animals: Syngeneic models if animal cell lines are inoculated in immunocompetent animals or xenograft if human cell lines are inoculated in immunosuppressed animals; (iii) genetically engineered animals; (iv) induce cancer development in the mammary gland through the administration of a specific carcinogenic compound. An appropriate rat model of mammary carcinogenesis should fulfill some requirements, specifically [7, 13]: (i) It should exhibit histopathological features and genetic alterations similar to those described in woman’s breast cancer; (ii) it should develop through a sequential flow of the different stages of carcinogenesis (initiation, promotion, progression, and metastization) and exhibit pre-neoplastic and neoplastic lesions; (iii) the tumors should develop specifically from the mammary gland tissue; (iv) a higher incidence (>60%) should be observed in a relatively short time (latency period lower than 6 months); and (v) the induction method should be easily reproducible by other researchers.

The knowledge of all steps from cancer induction until mammary tumor collection is essential for the success of the rat model of mammary cancer. Although several rat strains are available for experimental assays of chemically-induced mammary carcinogenesis, the Sprague-Dawley and Wistar outbred strains are more sensitive to chemical carcinogens when compared with other strains, such as Marshall and August rats [7]. Among these, the Sprague-Dawley strain is considered the most sensitive one, being the most frequently used in experimental assays of chemically-induced mammary carcinogenesis [14].

In this review, we provide the researchers with a detailed step-by-step guide to implement a rat model of mammary cancer, based on our wide experience in this field, to obtain the best results, the maximum throughput of each experiment, and easy comparison among researches.

## Chemical Carcinogens to Induce Mammary Tumors

A chemical carcinogen is any compound able to induce cancer development in an animal [15, 16]. Considering that mammary carcinogenesis induction should be simple, relatively fast, and safe for the manipulator [17], until now, only two chemical carcinogenic compounds gather these characteristics: 7,12-dimethylbenz[a]-anthracene (DMBA) and *N*-methyl-*N*-nitrosourea (MNU) [7, 13]. Once mammary cancer development is hormone-dependent, the induction rate will depend on the female rats’ age. The maximum incidence is reached when the carcinogen is administered between 45 and 60 days of age, which matches the animals’ sexual maturity and the differentiation of the terminal end buds of the mammary gland [18].

### 7,12-Dimethylbenz[a]-anthracene

7,12-dimethylbenz[a]-anthracene is a classical polycyclic aromatic hydrocarbon [19]. A single administration of this carcinogen dissolved in sesame or sunflower oil, by gavage at 50–56 days of age, in a dose ranging from 10 to 100 mg/kg, induces the development of mammary tumors [20]. To exert its carcinogenic effects, DMBA must be previously bioactivated by the cytochrome P-450/P1-450 monooxygenase enzyme systems, mainly located in the liver. The metabolites are mono- and dihydroxymethyl, and they can also be metabolized to their corresponding dihydrodiols, phenols, and other oxidation products [21]. The produced epoxides interact with the DNA generating the transversions A: T for T: A and G: C for T: A, through two mechanisms: The formation of adducts DMBA-adenine and DMBA-guanine, and the loss of purines by spontaneous lysis of the complex between the DMBA epoxide and DNA purines target [22]. 7,12-dimethylbenz[a]-anthracene is more difficult to use when compared with other carcinogens, because it is oil soluble and must be administrated by gavage. This technique is associated with animal suffering and death.

### *N*-methyl-*N*-nitrosourea

The MNU is represented by the molecular formula C_2_H_5_N_3_O_2_ and has a molecular weight of 103.08 g/mol [23]. The MNU belongs to the group of alkylating agents. It transfers an alkyl group and reacts with nucleophilic nitrogen and oxygen atoms in the purine and pyrimidine bases and the phosphate group of DNA, producing a wide range of DNA adducts, namely, N^7^ and N^3^ alkylpurines and O^6^-alkylguanine and O^4^-alkylthymine [24]. Although O^6^-alkylguanine is a minor alkylation product, it is considered responsible for the mutagenic and carcinogenic actions of this compound [25]. O^6^-alkylguanine is produced by the alkylation of the DNA molecule at the O^6^ position of the guanine. This alteration in the DNA structure will create wrong nucleotide incorporation during DNA replication and consequently in RNA transcription, which has an important role in mutagenesis and carcinogenesis processes [26, 27]. Inversely to DMBA, MNU is a direct alkylating agent that does not require metabolic activation before interaction with DNA [28].

Following the administration of any of the carcinogens DMBA or MNU, female rats typically develop a wide spectrum of mammary neoplasia subtypes, including benign fibroadenomas, intraductal carcinomas, or invasive carcinomas, and the incidence of different neoplasia will vary due to different rat genetic backgrounds [29].

The MNU is commercialized as pale-yellow crystals that are soluble in water, polar organic solvents, alcohol, ether, acetone benzene, and chloroform [23]. It should be stored at −20°C, in the dark. The MNU dissolution is not easy, it takes more than 30 min and it is difficult to exceed the concentration of 10 mg/mL [30]. The stability of MNU in aqueous solutions is favored at a pH of 4 (half-life 125 h) and disfavored at a pH of 9 (half-life of 1 min and 48 s), at the temperature of 20°C [23]. Usually, MNU is dissolved in an aqueous vehicle acidified to a pH of about 5, and the solution should be used within 3 h after its preparation. *N*-methyl-*N*-nitrosourea has a half-life of approximately 30 min after its administration [30]. At high values of pH, the MNU solution is very reactive with water, facilitating decontamination [23].

**Figure-1:**
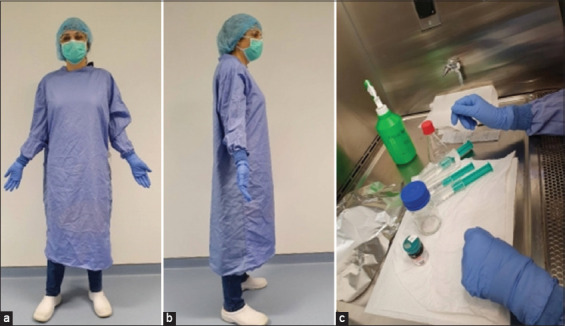
(a and b) Personal protective material used for the preparation and administration of carcinogens and (c) *N*-methyl-*N*-nitrosourea preparation in a fume cupboard. [Source: Figure prepared by the authors].

As potent carcinogenic agents, all operations involving the handling and the administration of MNU and DMBA should be carried out taking appropriate safety measures, such as: Using two pairs of gloves, a mask, an impermeable lab coat (Figure-1), and a laminar flow chamber reserved for its manipulations. The MNU may be destroyed by its mixture with saturated aqueous sodium bicarbonate [30, 31]. The waste should be eliminated as Type IV waste in proper containers, following European guidelines.

## Experimental Protocol

Before carrying out the experimental protocols, it is necessary to obtain authorization from the Institutional Ethics Committee and National Competent Authority, because only with them the compliance with the legal precepts can be done and, in the future, the publication of the results be guaranteed.

Although we have already performed the experimental induction of mammary cancer with DMBA in female rats, we generally use MNU, not only because MNU administration is easier and less aggressive for animals when compared with DMBA administration, but also because the mammary carcinomas MNU-induced have a higher aggressiveness and worse prognostic when compared with those DMBA-induced. In this way, the rat model of MNU-induced mammary tumors is advised in experimental protocols aiming to study more aggressive mammary tumors [32].

**Figure-2:**
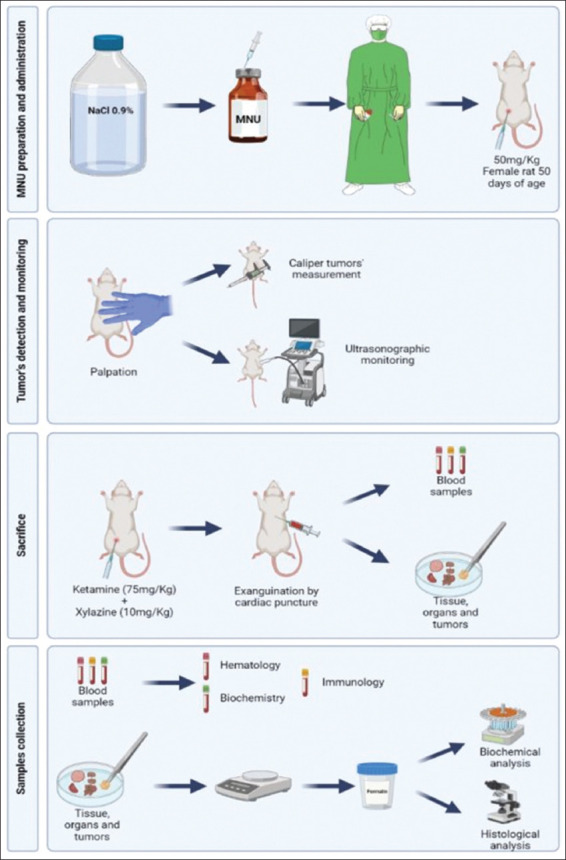
Schematic representation of a protocol of mammary carcinogenesis *N*-methyl-*N*-nitrosourea-induced in female rats, starting with the preparation of the carcinogen agent, and finishing with the animals’ sacrifice and biochemical and histopath [Source: Figure prepared by the authors].

To induce mammary tumor development (Figure-2), MNU may be administered intraductally, intraperitoneally, subcutaneously, or intravenously. Intraperitoneal administration is the most often used route, and MNU administration to female Sprague-Dawley and Wistar rats, on 50 days of age, at a dose of 50 mg/kg is recommended [33, 34], and a 100% induction rate can be reached. When animals are not familiar with handling, it is recommended to use chemical restraint (sedation) to increase the safety of administration, for both the animal and manipulator. However, if the animals frequently and adapted to the handler, in our opinion, sedation is not necessary.

**Figure-3:**
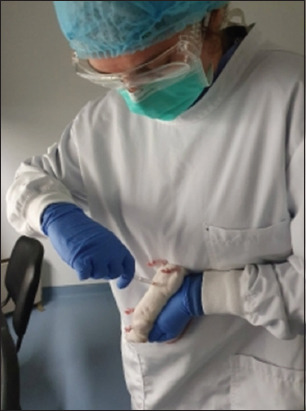
Intraperitoneal administration of *N*-methyl-*N*-nitrosourea in a female rat [Source: Figure prepared by the authors].

For the MNU intraperitoneal administration, the animals must be restrained in supine position by an experienced researcher, as shown in Figure-3. Normally, we used 27-gauge needles and syringes of 1 mL. To ensure that viscera are not punctured during intraperitoneal administration, we cutoff the tip of the needle cap, leaving out only the length of the bevel. The researcher must use a mask, protective glasses, two pairs of gloves, a lab coat, and a surgical cap. All materials involved in the preparation and administration of MNU must be disposed of as hazardous waste. Each geopolitical entity has its own legislation for the disposal of this type of waste, but it must always be complied with so that there is no contamination of people or the environment.

## Follow-up of Mammary Cancer Development

**Figure-4:**
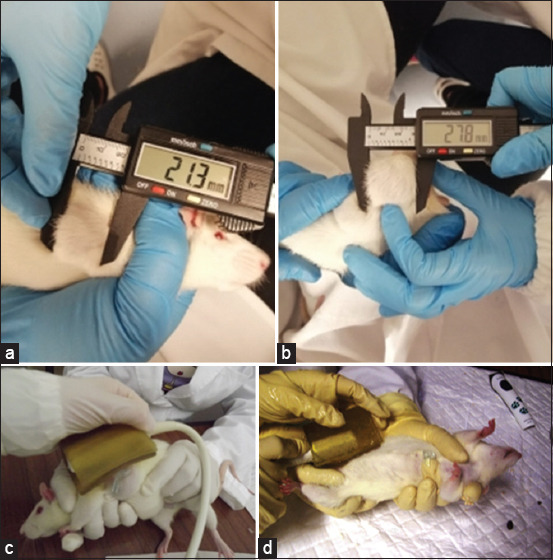
(a) Measuring the length and (b) width of (c and d) tumors with a digital electronic caliper and ultrasonographic monitoring of mammary tumor by ultrasonography. Sagittal and transverse scans can be performed, with B-mode. Power Doppler, B Flow and Contrast-enhanced ultrasound [Source: Figures prepared by the authors].

During the experiments, both mammary chains of each animal must be palpated for the detection of mammary tumor development According to our experience, the first mammary tumor can be detected between the 8^th^ and 12^th^ week after the carcinogen administration. According to our experience, an incidence of 100% was only reached in one experiment, 35 weeks after the MNU administration [35], probably due to the shorter duration of the other studies (18 and 19 weeks, respectively). Mammary tumors’ size can be monitored using a caliper (Figures-4a and b) or by ultrasonography using B-mode (Figures-4c and d). Mammary tumors seemed to have an oblate spheroid geometry. According to our experience, the most accurate volume calculation is obtained using the formula V = (W^2^ × L)/2 for caliper measurements and the formula V = (4/3) × π × (L/2) × (L/2) × (D/2) for ultrasonography measurements, where V is tumor volume, W is tumor width, L is tumor length, and D is tumor depth [36].

**Figure-5:**
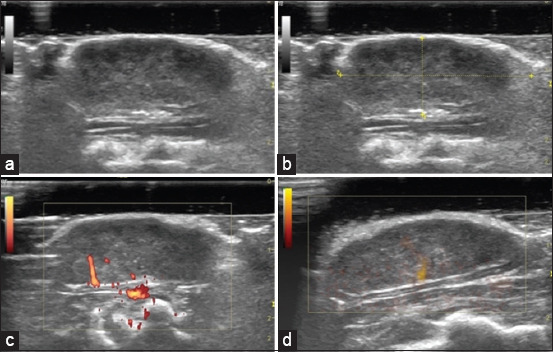
(a and b) Monitoring of mammary tumors by ultrasonography, using B-mode, (c and d) Power Doppler and B Flow. (b) The tumor length and depth can be measured using the using electronic cursors integrated into the ultrasound machine and set at the borders of the tumor [Source: Figure prepared by the authors].

Mammary tumors’ vascularization may be assessed using Power Doppler, B Flow, Pulsed Doppler, and Contrast-enhanced ultrasound (CEUS) [37–39] (Figure-5). The ultrasonographic examination may be performed with awake animals if previously adapted to the handling, or require sedation. Usually, our animals are frequently handled, and we perform the ultrasonographic examination without any sedation. However, if necessary, the animals should be sedated by administering ketamine (37.5 mg/kg) and xylazine (5 mg/kg) intraperitoneally. During the examination, the animals should be restrained in the supine position, and the examination is made as quickly as possible by an experienced researcher. A standoff pad is usually used to improve the quality of ultrasonographic images of mammary tumors. Considering the rat size, a high-frequency ultrasound probe (10–12 MHz) was used by our research team. Using B-mode can be obtained images of mammary tumors in sagittal planes and the length and depth of each tumor may be measured using electronic cursors integrated into the ultrasound machine and set at the borders of the tumor (Figure-5b). The tumors’ vascularization can be addressed using Power Doppler and B Flow modes, by quantifying the color pixels density (CPD) in Adobe Photoshop or GNU Imaging Manipulation Program (Kimball and Mattis, USA) (CPD (%) = the number of colored pixels in the tumor/number of total pixels of the tumor) [40]. Moreover, the tumors’ vascularization can be assessed by CEUS. In our protocols, we injected 0.1 mL of the contrast agent SonoVue (Bracco, Italy) into the tail vein after its cannulation, followed by a flush of 1 mL of saline solution, with no risks for the animal. The use of contrast allows visualization of small vessels, not detected by B Flow or Doppler. Quantitative analysis of the CEUS images can be performed using the time-intensity curve analysis of the ultrasound apparatus [41]. According to our experience, ovoid region of interest can be drawn in the most enhanced/vascularized area of each mammary tumor.

## Data Collection

### Humane endpoint evaluation throughout the experiments

**Figure-6:**
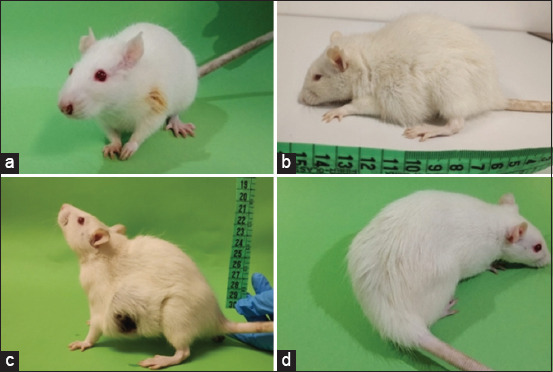
(a) Examples of humane endpoints alterations observed in our studies such as chromodachryorrhea, (b) hunched posture, (c) tumor ulceration on macroscopic evaluation, and (d) lack of grooming [Source: Figure prepared by the authors].

According to the European Guidelines, it is obligatory to monitor the animals’ welfare during experiment protocols. Researchers must improve their methods and put humane endpoints into place when there is the possibility of causing pain or suffering to laboratory animals. To do this, we suggest using the table of humane endpoints previously described by Faustino-Rocha *et al*. [42] for this model. According to this publication, several parameters should be monitored during the experiments, such as general appearance and mental status, body condition, body weight, food and water intake, posture, coat and grooming, mucosal, eyes, ears, and whiskers, and mental status, behavior (response to external stimuli), clinical parameters (hydration status, respiratory and heart rate, body temperature, hematocrit, and urine specific gravity) and mammary tumors’ location, macroscopic appearance, and burden. A score from 0 to 3 is attributed to each parameter. A sum equal to or higher than four indicates sacrifice. We also suggest considering as severe the following changes: weight loss above 20%, severe anemia, moribund or comatous mental status, tumors’ interference with the animal’s ability to eat and drink, tumors contact with the cage floor or even tumor burden above 10% of the animal body weight, and tumor ulceration. The animal should be immediately sacrificed if one of the described parameters is identified. We also suggest that animals should be monitored by the same researcher once a day. Some changes observed in the described parameters are displayed in Figure-6, such as chromodachryorrhea, hunched posture, tumor ulceration, and lack of grooming.

### Body weight determination

**Figure-7:**
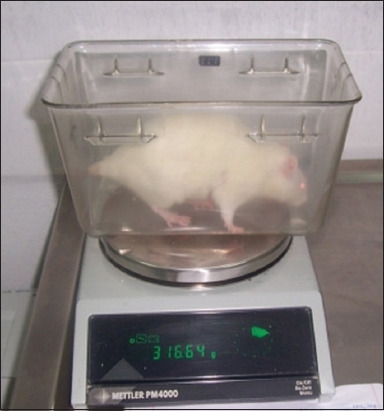
Animal weighing using a top-loading scale [Source: Figure prepared by the authors].

The gain or loss of body weight provides information concerning animals’ health status that is crucial to implement humane endpoints and guarantee animal welfare. In healthy rodents, body weight increases in two stages: During growth and post-maturity growth [43]. According to our experience, animals should be observed every day and weighed once a week using a top-loading scale (Figure-7). To obtain animals’ accurate body weight, tumor weight must be subtracted from total body weight at the end of the experiment (final body weight), because the animals can develop tumors weighing more than 30 g. If this subtraction is not made, it is mistakenly assumed that the animals’ weight has increased.

### Food and water consumption monitoring

The metabolic rate and food intake combination determines energy balance [44]. This depends on the ambient temperature, which determines the energy cost spent on thermoregulation, which is a determining factor in daily food intake [45]. The mean food and water intake assessment should be performed for both control and induced groups. According to our experience, the animal food and water intake was higher in the induced groups, when compared with the control group. Small variations in food and water intake may be related to the animals’ strain and age and duration of the experimental protocol.

### Sample collection during the experiment and animal sacrifice

**Figure-8:**
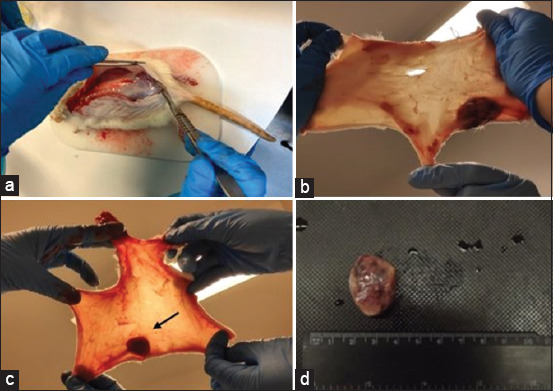
Performing of scalping technique and searching for small mammary tumors by examining the scalp under a light (arrow) [Source: Figure prepared by the authors].

During the experimental protocols, blood samples can be collected from the tail vein; urine and feces samples can be collected using metabolic cages. Animal sacrifice should be done according to the guidelines for laboratory animal use. Normally, we used an anesthetic overdose of ketamine and xylazine, but other methods should be employed. When anesthetized, the animals are shaved using a clipper machine. This procedure is essential for observing skin in more detail and for sample processing. After shaving, blood samples should be collected directly from the heart by cardiac puncture, and properly processed for hematological and biochemical determination. Afterward, female rats should be placed in the prone position and scalped, making an incision in the skin of the dorsal median line and a circular incision around each of the antebrachiocarpal and tibiotarsal joints. The skin must be removed like a coat, and carefully observed under light to detect small mammary tumors not previously detected by palpation (Figure-8). In our experience, by observing the skin under light, we usually have identified some tumors in the mammary gland unnoticed by palpation. Hence, scalping is critical for accurate knowledge of the number of mammary tumors induced by the carcinogen. In trials where treatments for mammary cancer are evaluated, not performing this procedure may affect the correct evaluation of the results, once the real number of induced tumors is unknown. Mammary tumors should be collected, weighed, measured using a caliper or a ruler, and their volume can be determined by water displacement (Figure-8).

In our research, we always collect all internal organs (liver, kidneys, lungs, heart, spleen, ovaries, and uterus), and then weigh and process them to understand the systemic influence of carcinogen administration. Due to these procedures, we have already identified lung metastases from mammary cancer.

### Mammary tumors’ histological analysis

Histological analysis is an essential step in understanding the induced spectrum of mammary lesions and assessing the efficacy of treatments. After collection and prior fixation, an incision should be made in the tumors to guarantee proper fixation. The tumors should be immersed in 10% phosphate-buffered formaldehyde for at least 24 h. Part of the tumor should be frozen at −80°C to perform biochemical determination. If intended to do an immunohistochemical study of the tumors, rat-specific antibodies should be used.

Then, fixated tumors should be cut, processed, and embedded in paraffin, and 3 mm-thick trimmed sections stained with hematoxylin and eosin (H&E) for histopathological evaluation. Histopathological analysis should be performed by two blinded histopathologists, and according to the guidelines for the classification of rat mammary tumors previously established by Russo and Russo [19].

We have observed that some authors classify induced tumors using the woman classification, but this is not correct and does not allow a correct analysis and comparison of the results among published papers that used animal models of mammary cancer.

**Figure-9:**
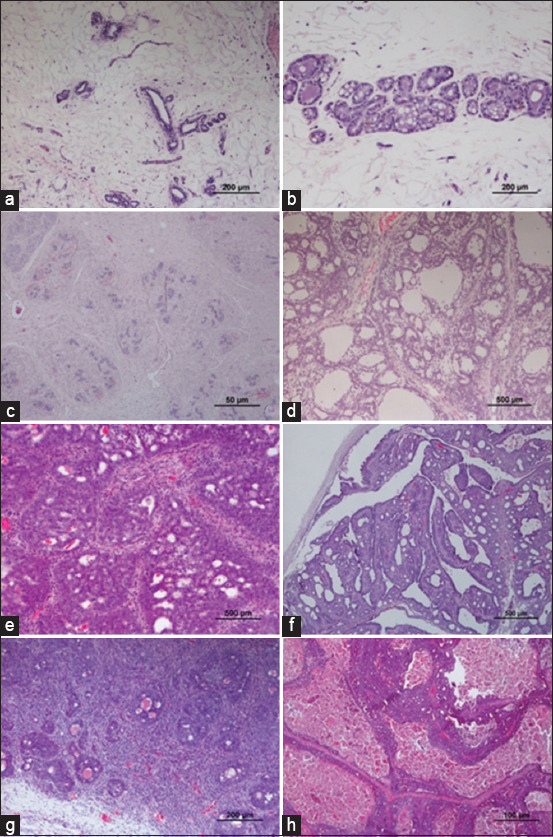
(a) Normal mammary gland; (b) mammary hyperplasia; (c) mammary fibroadenoma, (d) mammary adenoma; (e) mammary carcinoma; (f) mammary intraductal papillary carcinoma; (g) mammary invasive carcinoma; and (h) mammary comedo carcinoma (Hematoxylin and eosin) [Source: Figures prepared by the authors].

A wide spectrum of mammary lesions from benign, pre-neoplastic, and malignant lesions can be identified in the histopathological analysis, with papillary and cribriform non-invasive carcinoma the most common patterns. Invasive comedocarcinoma is the most aggressive mammary lesion identified in the MNU model of mammary cancer (Figure-9). Because rodents have dyssynchronous mammary development, more than one pathway may be identified in an individual animal after carcinogen exposure [46].

## Conclusion

Pre-clinical studies in animal models of mammary cancer constitute an important tool for the investigation of cancer biopathology and search for new and more effective therapeutic approaches. Thus, based on our experience, in this paper, we provide a detailed description of the various steps to be followed in the rat model of mammary cancer, presenting, in detail, the dose and route of the administration of the chemical compound, the cancer detection and monitoring by ultrasonography, the animals’ welfare monitoring and application of humane endpoints, the method for animals’ sacrifice and sample collection, and histological analysis of mammary tumors. We are confident that following the steps described in our manuscript, the researchers will successfully implement the rat model of chemically-induced mammary carcinogenesis, obtain the best results, maximum throughput of each experiment, and easily compare the results among researchers.

## Authors’ Contributions

AIF and PAO: Conceptualized the review. JS, AIF, and PAO: Searched for the literature. JS and AIF: Drafted the manuscript. JAD and PAO: Reviewed the manuscript. All authors have read, reviewed, and approved the final manuscript.
